# Feasibility of multi‐energy CT and fat fraction MRI for characterizing active bone marrow response to pelvic external beam radiation therapy

**DOI:** 10.1002/acm2.70794

**Published:** 2026-09-17

**Authors:** Carolyn Eckrich, Kristin Bradley, Bethany Anderson, Diego Hernando, Jessica Miller, Jainil Shah, Patrick Wohlfahrt, Michael Lawless

**Affiliations:** ^1^ Department of Radiation Medicine University of Wisconsin‐Madison Madison Wisconsin USA; ^2^ Department of Medical Physics University of Wisconsin‐Madison Madison Wisconsin USA; ^3^ Cancer Therapy Imaging Siemens Healthineers, Varian Cary North Carolina USA; ^4^ Cancer Therapy Imaging Siemens Healthineers, Varian Forchheim Germany

**Keywords:** active bone marrow, fat fraction MRI, multi‐energy CT

## Abstract

**Background:**

Pelvic external beam radiation therapy (EBRT), frequently exposes a substantial fraction of active bone marrow (ABM), leading to radiation‐induced marrow suppression and associated hematologic toxicity. Accurate delineation and quantitative assessment of ABM are therefore essential to enable marrow‐sparing radiotherapy strategies.

**Purpose:**

To identify and quantify changes in ABM after EBRT using multi‐energy CT (MECT) and fat fraction MRI (FFMRI).

**Methods:**

Patients with gynecological malignancies underwent MECT and FFMRI pre‐ and post‐radiation therapy. ABM was delineated on both imaging modalities using thresholding where ABM was defined as voxels with CT numbers above the mean in MECT‐derived virtual non‐calcium (VNCa) datasets and voxels with percent fat fraction (%FF) below the mean on the FFMRI. ABM volumes and mean HU/%FF values were evaluated before and after treatment. A Wilcoxon‐signed rank test was computed to compare paired MECT versus FFMRI ABM volume percent changes. Changes in ABM as a function of dose were also evaluated. A paired t‐test was computed for each dose gradient (significant *p* < 0.05).

**Results:**

The average Dice Similarity Coefficient (DSC) between the MECT and FFMRI pre‐treatment ABM volumes was 0.57 (range: 0.50–0.68). The mean volumetric change in ABM volume between pre‐ and post‐treatment scans was −37%±15% (range −57% to −12%) and −66%±27% (range 8% to −94%) for MECT and FFMRI, respectively. All paired patients demonstrated greater ABM volume reduction with FFMRI than with MECT, and this difference was statistically significant (*p* = 0.0078). ABM changes were observed as a function of dose: A decrease in mean CT numbers and an increase in %FF corresponds to an increase in dose. CT number decreased significantly at doses ≥10 Gy (*p* ≤ 0.005), while %FF increased significantly across all dose gradients (*p* ≤ 0.0017), with both metrics showing strong dose‑response behavior at higher doses (*p* < 10^−^^6^).

**Conclusions:**

This feasibility study demonstrates the utility of using MECT and FFMRI for delineating ABM in patients receiving pelvic EBRT. These imaging modalities showed good agreement in characterizing ABM volumes changes and radiation‐induced marrow suppression. These findings support further investigation of ABM segmentation into the RT‐planning workflow to facilitate marrow‐sparing strategies.

## 1 INTRODUCTION

Bone marrow (BM) is the soft tissue in the medullary cavities of bones, which is composed of active (red) marrow and inactive (yellow) marrow. Active bone marrow (ABM) is hematopoietically active and largely located in the axial skeleton (pelvis, spine, skull).^1^ In contrast, inactive bone marrow is characterized by a high adipocyte content and minimal hematopoietic activity.^2^ During external beam radiation therapy (EBRT), BM and circulating blood cells are exposed to radiation doses which is associated with an increased risk of developing hematologic toxicities (HT).^3^, ^4^

A comprehensive understanding of ABM distribution is critical to minimizing the risk of HT in patients undergoing combined chemoradiotherapy for pelvic malignancies. Patients with locally advanced cervical cancer are often treated with chemoradiation, which can increase the risk of HT. When a patient experiences HT, chemotherapy may need to be delayed or omitted, which can negatively impact treatment outcomes.^5^, ^6^, ^7^ Previous studies have identified relationships between BM radiation dose‐volume metrics and HT in patients, suggesting that BM sparing treatment planning techniques could reduce HT.^8^, ^9^, ^10^, ^11^ Identifying and sparing regions of ABM, as opposed to the entire BM compartment, could provide a similar reduction in HT while imposing less of a constraint on the planning process.^5^, ^10^ Identifying IBM and ABM is challenging and currently the most established means of evaluating BM is through bone marrow biopsies; however, this process is invasive, painful, and requires sedation or general anesthesia.^12^ Although a biopsy offers definitive insight into the sampled tissue, its findings are limited to that specific location. It does not provide information about marrow characteristics elsewhere, making it unsuitable for radiation dosimetry.

Multi‐energy CT (MECT) is a promising modality for ABM identification through its ability to perform material decomposition. This concept was originally introduced in foundational studies by Alvarez and Macovski^13^ and later expanded upon by Kalendar et al.^14^ in the 1970s and 1980s. Recent advancements in CT technology have significantly improved the accessibility and cost effectiveness of MECT, facilitating its integration into clinical and research settings. MECT allows for the use of material decomposition algorithms to subtract calcium from images, thereby generating virtual non‐calcium (VNCa) images. D'Angelo et al.^15^ provided a comprehensive overview of the principles underlying VNCa image generation and its emerging clinical applications, including the removal of vascular calcifications and the evaluation of BM edema. Following calcium subtraction within BM regions, variations in CT numbers given in Hounsfield units (HU) reflect differences in the density of marrow tissues. ABM, characterized by higher perfusion and blood content, is expected to exhibit higher CT numbers on VNCa images compared to IBM, which predominantly consists of low‐density adipose tissue. This concept was benchmarked in standard and anthropomorphic phantoms,^16^ and was further validated against histologic samples^17^ and in human cadavers.^18^ MECT VNCa images have also been used to monitor changes within the bone marrow due to cancer therapy.^19^, ^20^

In a review of BM adiposity research and associated methodologies, Tratwal et al.^21^ identified MRI as the most effective modality for the in vivo assessment of BM adiposity. Reeder et al.^22^ introduced an MRI method based on chemical shift encoding for water‐fat separation (proton density fat fraction magnetic resonance imaging, FFMRI), to enable quantitative assessment of tissue fat content. FFMRI is considered to be the gold standard for assessing bone marrow adiposity and has been benchmarked against histological samples.^21^, ^22^, ^23^, ^24^, ^25^ In the context of EBRT, FFMRI has been used as a surrogate measure of ABM.^11^, ^26^, ^27^ This MRI technique allows for the quantitative assessment of fat content within each voxel. In FFMRI, each voxel value represents the percentage of fat composition. A voxel value of 0 corresponds to 0% fat, indicating a composition of 100% water, whereas a value of 100 denotes 100% fat and no water content. High fat fraction values are indicative of less active, adipose‐rich marrow, while lower fat fractions suggest more active marrow, characterized by increased perfusion and reduced fat content. MR scanners are becoming more prevalent in radiation oncology clinics and could be used to identify low adiposity regions of the marrow for planning and patient monitoring purposes.

^18^F‐fluorothymidine positron emission tomography (FLT‐PET) is widely regarded as the gold standard for imaging active bone marrow, as it directly measures proliferative marrow activity by targeting thymidine kinase in replicating cells.^28^, ^29^, ^30^ Studies have consistently demonstrated that FLT uptake correlates strongly with histologic assessments of hematopoietic activity, establishing its clinical value in noninvasive bone marrow evaluation.^31^ However, FLT‐PET is not yet FDA‐approved, which limits its clinical use. ABM, identifiable on PET imaging using tracers such as FLT and ^18^F‐fluorodeoxyglucose (FDG), has become a focus for reducing acute HT during pelvic chemoradiotherapy. A pioneering methodology by McGuire et al. utilized FLT PET to delineate highly active marrow subregions and incorporated these PET‐defined regions as avoidance areas in IMRT planning, achieving reductions in volumetric doses to ABM without compromising target or OAR coverage.^9^ Building upon PET‐defined marrow mapping, Mell et al.^32^ demonstrated that the absolute volume of FDG‐PET defined pelvic ABM spared at specific dose levels strongly predicted the incidence of grade 3–4 HT in anal cancer patients undergoing chemoradiation.

The objective of this study is to evaluate the feasibility of using MECT and FFMRI to identify and quantify ABM changes in patients undergoing pelvic EBRT. Unlike prior approaches that rely on PET imaging—particularly FLT‐PET, which remains costly and not widely available—our work introduces two clinically accessible, noninvasive imaging modalities that can be integrated into routine workflows without the logistical and regulatory barriers of PET. This study establishes a foundation for biologically informed treatment planning using imaging modalities that are already available in most radiation oncology clinics. This represents a novel step toward practical marrow‐sparing strategies that could reduce hematologic toxicity, maintain chemotherapy compliance, and ultimately improve patient outcomes in gynecologic cancer care. FFMRI and MECT have been shown to be an effective means of measuring the adiposity of bone marrow, and previous work by the authors benchmarked FFMRI and MECT against FLT‐PET for identification of proliferative ABM in a swine model.^20^ Thus, in this work, adiposity as measured by FFMRI and MECT was used as a surrogate for ABM. This work also investigated changes in ABM volumes after EBRT using FLT‐PET, MECT, and FFMRI at various timepoints in a swine model.^33^

## 2 MATERIALS AND METHODS

### 2.1 Patients

With approval from the institutional review board (IRB), a prospective study was performed for patients who were treated with gynecological malignancies at our institution using radiation or chemoradiation therapy between 2023 and 2025. Patient characteristics are shown in Table 1.

**TABLE 1:** Patient characteristics.

Characteristics	
Number of patients	15
Median age, years (range)	64 (34–71)
Diagnosis site	
Cervical	4
Endometrial	5
Vaginal	3
Vulvar	3
Brachytherapy	6
Chemotherapy	
Cisplatin	9
Carbo/Taxol	2
None	4
Imaging	
MECT	10
FFMRI	15

### 2.2 Imaging modalities

MECT images were acquired on a Siemens SOMATOM Definition Edge scanner (Siemens Healthineers, Forchheim, Germany) using an 80 kVp/140 kVp sequential scanning technique, Dual Spiral (DS). The syngo.via software (version VB60, Siemens Healthineers, Forchheim, Germany) employs a three‐material decomposition. A dual‐energy ratio of 1.57 was manually entered into the software following the measurements performed in phantom by Miller et al.^34^ Basis materials of calcium, water, and fat were used to generate virtual non‐calcium (VNCa) images. The DS VNCa images represent a 120 kVp‐equivalent image in the absence of calcium, and each voxel is displayed in HU. A standard 120 kVp‐equivalent “mixed” image, which did not have the calcium removed, was also created in syngo.via. The mixed images were used for the creation of the patient's external beam radiation therapy plan. Patients were immobilized following our institution's standard practice for pelvis patients.

All MRI acquisitions were acquired using a 1.5‐T SIGNA Artist MRI system (GE Medical Systems). The patient was setup in the treatment position with immobilization devices to closely match the CT scan positioning as possible. All acquisitions included an axial T2*‐corrected chemical‐shift encoded IDEAL‐IQ acquisition for fat fraction estimation. The IDEAL IQ acquisition produces Fat, Water, R2* and Fat Fraction images.

Ten patients received MECT and fifteen patients received FFMRI images prior to treatment and 3–6 months post‐completion of their radiation therapy treatment course at a routine follow‐up visit.

### 2.3 Image analysis

All images were analyzed with MIM Maestro software (v7.2.8). The relevant bony anatomy of the spine and pelvis was segmented on the 120 kVp‐equivalent mixed CT image using the TotalSegmentator open‐source AI contouring software.^35^, ^36^ This volume was contracted by three millimeters to exclude the cortical bone to obtain a contour of the bone marrow. The contour was further edited manually to remove remaining cortical bone. The bone marrow contour was propagated to the FFMRI images using a piecewise rigid registration strategy, in which each vertebral body or pelvic bone (e.g., L4, L5, right femur) was independently rigidly registered between MECT and FFMRI. The resulting transformation was applied only to the corresponding bony structure. To define ABM volume, a relative, patient‐specific threshold was applied within the bone marrow contour. Analogous to prior studies using PET‐based ABM segmentation, a mean‐based thresholding approach was used.^8^, ^20^, ^37^, ^38^, ^39^, ^40^, ^41^, ^42^, ^43^ On MECT images, ABM was defined on pre‐treatment scans as voxels with CT numbers above the mean value within the marrow region of MECT‐derived VNCa images. On FFMRI images, ABM was defined as voxels with %FF below the mean value within the marrow region. This mean‐based thresholding approach was used to characterize the relative distribution of higher‐density and lower‐fat marrow within each patient rather than to define an absolute biological marrow volume. The relative threshold used will encompass the most active region of the patient's marrow. For post‐treatment images, the same pre‐treatment ABM threshold was applied to assess treatment‐induced changes in ABM volume and mean CT number/%FF. The Dice similarity coefficient (DSC) was calculated between pre‐treatment ABM volumes derived from MECT and FFMRI. ABM volumes were segmented pre‐ and post‐treatment for each modality, and for patients with both MECT and FFMRI, paired differences in percent volume change between modalities were evaluated using a two‐sided Wilcoxon signed‐rank test with statistical significance defined as *p* < 0.05.

### 2.4 Volume response to dose

To evaluate the effect of radiation dose on ABM volume loss, each patient's clinical IMRT treatment plan was imported into the research workflow. Using the dose distribution from the clinical plan, ABM volumes were stratified into dose bins (e.g., 5–10 Gy, 10–15 Gy, 15–20 Gy, 30–40 Gy, and 40+ Gy). For each bin, the pre‐treatment ABM volume was recorded, and the corresponding post‐treatment imaging was used to quantify residual ABM within the same spatial regions. This approach enabled assessment of dose‐dependent marrow‐suppression by correlating ABM volume changes with delivered radiation dose levels. For each dose bin, paired comparisons were performed using a two‐tailed paired t‐test to assess pre‐ versus post‐treatment changes. Statistical significance was defined as *p* < 0.05. Only doses from the patients’ pelvic EBRT plans were included in this analysis. Any other radiation therapy treatment (brachytherapy or EBRT) were not included in this analysis.

## 3 RESULTS

Of the patient cohort, 8 total patients received the full imaging regimen: pre‐ and post‐treatment MECT and FFMRI images. Ten patients received the pre‐treatment MECT imaging while only 9 received post‐treatment imaging. For FFMRI, one patient had prominent imaging artifacts on the post‐treatment FFMRI, which was not used for analysis in this study.

**FIGURE 1:**
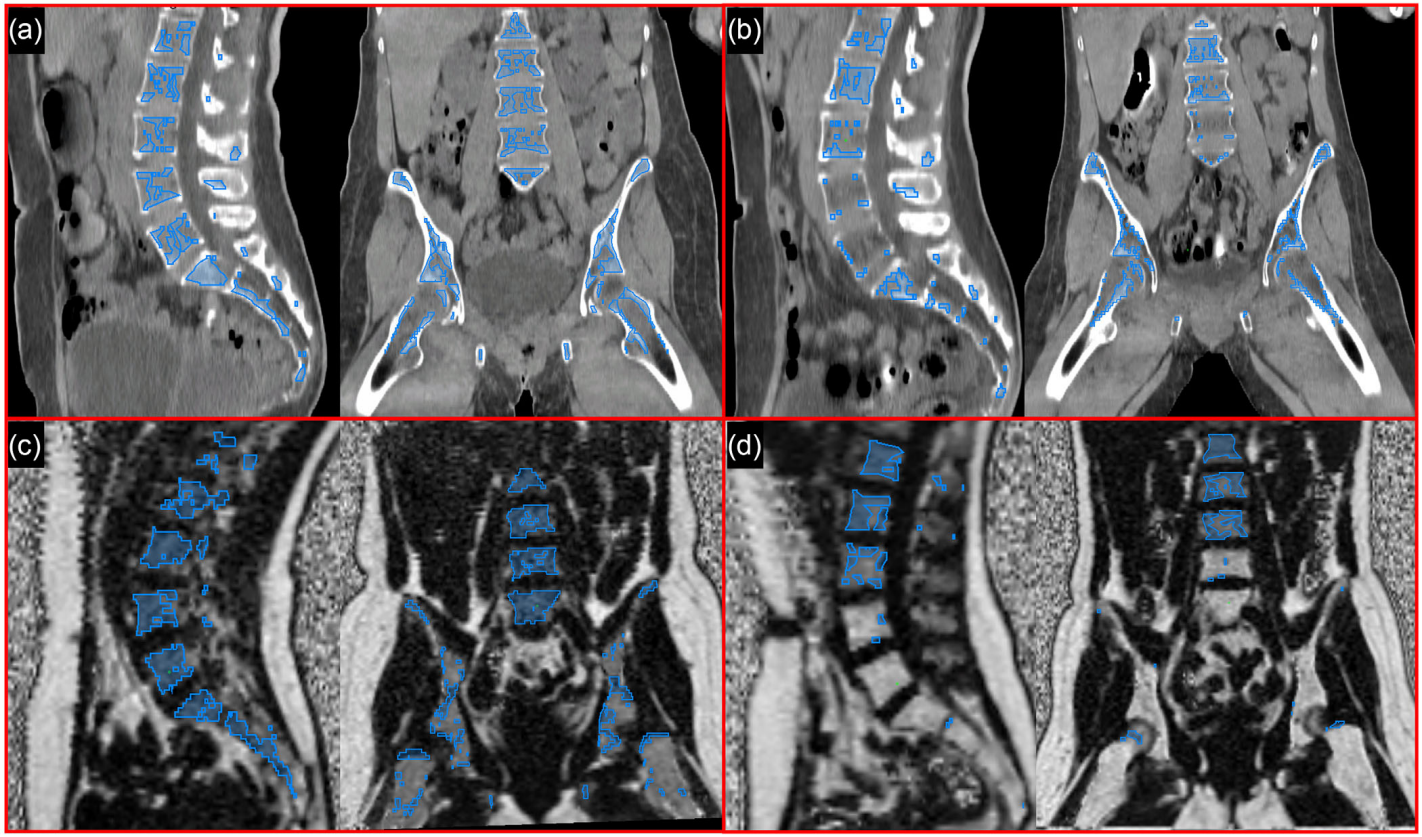
Example of active bone marrow contours (shown in blue) on (a) pre‐treatment virtual non‐calcium (VNCa) image derived from multi‐energy CT, (b) post‐treatment VNCa, (c) pre‐treatment fat fraction MRI (FFMRI), and (d) post‐treatment FFMRI.

**FIGURE 2:**
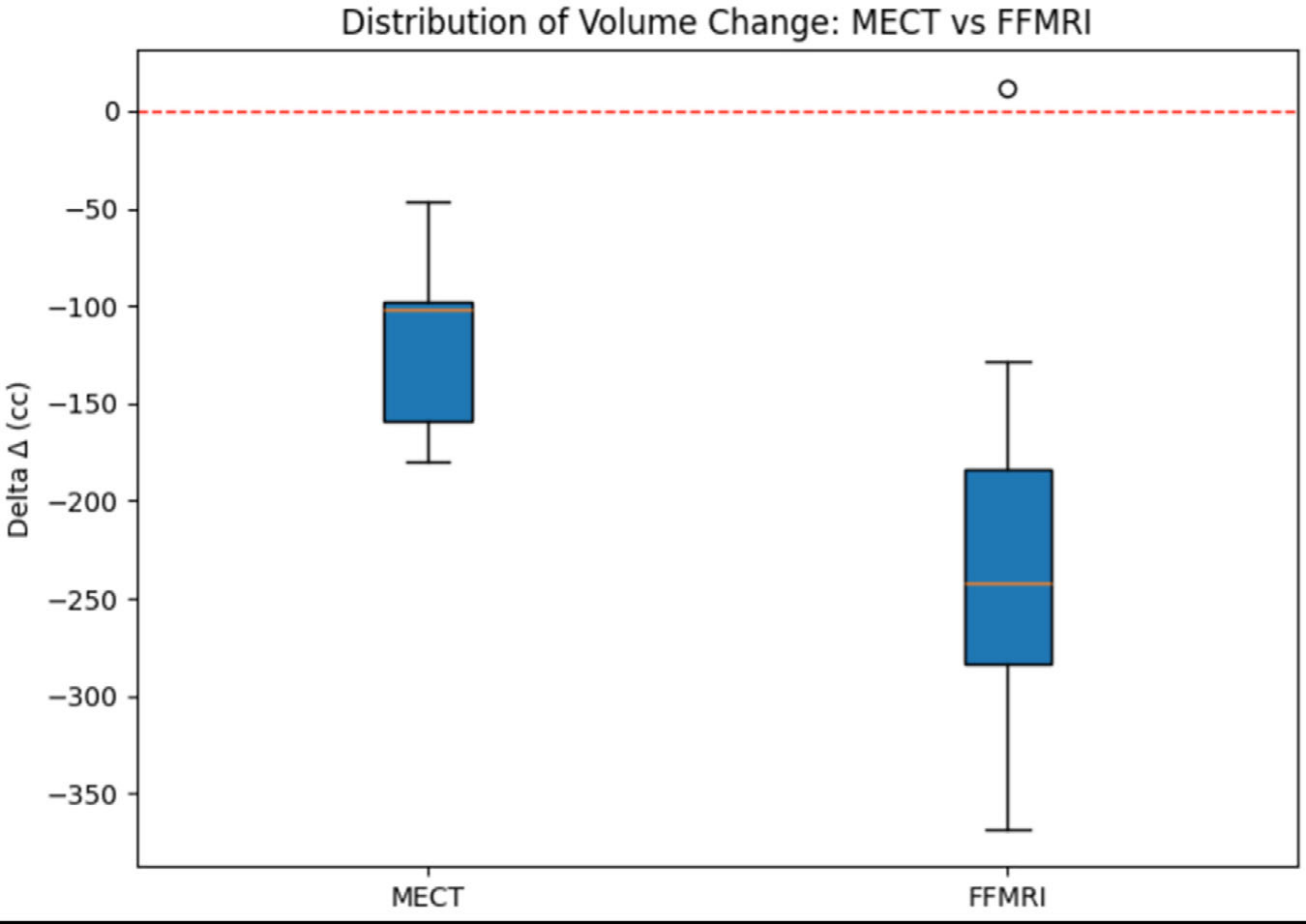
Distribution of pre‐ to post‐treatment active bone marrow volume change (**Δ) **measured on multi‐energy CT (MECT) and fat fraction MRI (FFMRI) across 9 MECT and 14 FFMRI patients. The mean **Δ **for MECT was −116.0 cc (−37%), whereas FFMRI showed a substantially larger mean **Δ **of −225.0 cc (−66%).

Figure 1 shows pre‐ and post‐treatment MECT and FFMRI scans of the same patient with ABM contours on each respective scan. The mean CT number threshold for BM was 33.27 HU for the 10 patients that received pre‐treatment MECT imaging for MECT and 57.66 %FF for the 15 patients that received pre‐treatment FFMRI. The pre‐treatment ABM volumes were compared between MECT and FFMRI using the Dice similarity coefficient (DSC). The average DSC between the volumes was 0.57 (range 0.50–0.68) across 9 patients with paired pre‐treatment MECT and FFMRI imaging. The images show an overall decrease in ABM volume between the pre‐ and post‐treatment scans. The mean volumetric change in ABM volume across the patients (*n* = 9 for MECT, *n* = 14 for FFMRI analysis) was −37% ± 15% and −66% ± 27% for MECT and FFMRI, respectively. The mean change (**Δ) **in ABM volume between pre‐ and post‐treatment images is shown in Figure 2 for MECT and FFMRI, respectively.

**FIGURE 3:**
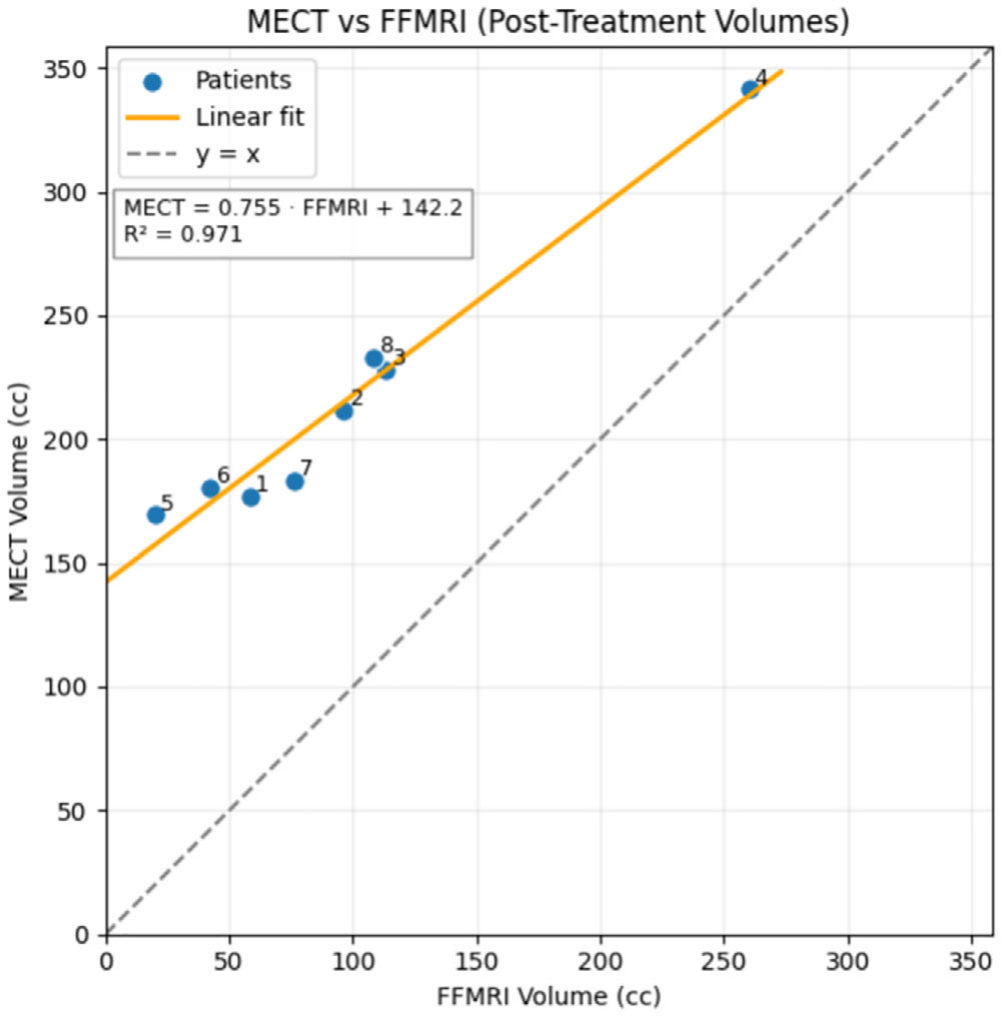
Post‑treatment MECT volume is plotted as a function of FFMRI volume for eight patients who underwent pre‐ and post‐treatment MECT and FFMRI imaging. The solid orange line represents the linear regression fit, while the gray dashed line indicates the line of equality (y = x).

On average, MECT produced ABM volumes that were 107% larger than those derived from FFMRI on post‐treatment images. Figure 3 shows post‐treatment MECT ABM volumes as a function of FFMRI for 8 patients.

Among patients who received both imaging modalities (*n* = 8), the observed percent ABM volume reduction was consistently greater as seen on FFMRI than MECT (Wilcoxon signed‐rank test, W = 0, *p* = 0.0078).

### 3.1 ABM changes as a function of dose

**FIGURE 4:**
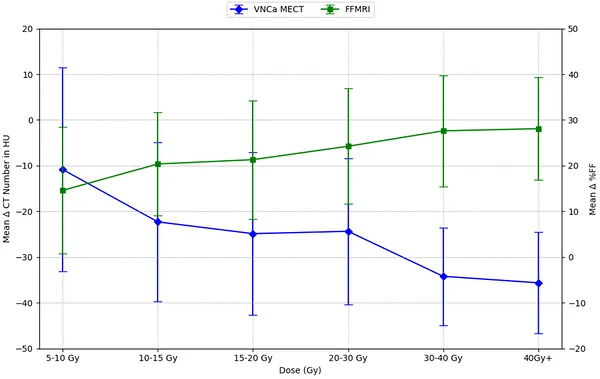
Mean difference (Δ) in active bone marrow values for multi‐energy CT (MECT) derived virtual non‐calcium (VNCa) images (CT number in HU, *n* = 9 for each dose bin) and fat fraction MRI (FFMRI, %FF, *n* = 14 for each dose bin) with respect to pre‐treatment at different dose gradients.

Figure 4 shows the average change relative to the pre‐treatment image (Δ) for the VNCa MECT and the FFMRI scans as a function of EBRT dose received. For MECT, the results show that regions of the marrow that received higher doses experienced larger decreases in CT numbers (*n* = 9). Similarly, for FFMRI, the results demonstrate a larger increase in %FF with increasing dose (*n* = 14). For the pre‐treatment images, the reported CT value represents the mean of patient‐specific mean BM CT numbers, which was 33.3 HU. Similarly, the mean of patient‐specific mean BM %FF for FFMRI was 57.7 %FF. Post‐treatment, the mean CT number decreased to 6.0 HU but increased to 75.8 %FF for FFMRI. Paired t‑tests revealed no statistically significant HU change in the 5–10 Gy dose bin (*p* = 0.18); however, significant decreases were observed at 10–15 Gy (*p* = 0.0049), 15–20 Gy (*p* = 0.0030), 20–30 Gy (*p* = 0.0018), 30–40 Gy (*p* = 1.1 × 10^−^^5^), and > 40 Gy (*p* = 1.2 × 10^−^^5^). In contrast, %FF demonstrated significant post‑treatment increases across all dose levels, beginning at 5–10 Gy (*p* = 0.0017) and strengthening with increasing dose, including 10–15 Gy (*p* = 1.4 × 10^−^^5^), 15–20 Gy (*p* = 3.4 × 10^−^^5^), 20–30 Gy (*p* = 7.0 × 10^−^^6^), 30–40 Gy (*p* = 1.2 × 10^−^^6^), and > 40 Gy (*p* = 3.6 × 10^−^^7^). A monotonic dose‐response relationship across dose bins was evaluated using Spearman rank correlation of bin‐averaged changes against dose bin midpoints. Mean ΔHU demonstrated a strong negative correlation with dose (ρ = ‐0.94, *p* = 0.0048), indicating progressively greater HU reduction with increasing dose. Mean Δ%FF demonstrated a strong positive correlation with dose (ρ = 1.0, *p* < 0.0001), consistent with increasing fat fraction at higher doses. No correction for multiple comparisons was applied.

## 4 DISCUSSION

This prospective study evaluated radiation‐associated changes in ABM volume using paired pre‐ and post‐treatment MECT and FFMRI. A substantial decrease in ABM volume was observed following treatment, with larger changes occurring in regions receiving higher dose. To our knowledge, this is the first prospective study to directly compare MECT and FFMRI for evaluating dose‐dependent changes in ABM in patients undergoing pelvic radiation therapy. Wang et al.^44^ reported on the correlation between changes to pelvic bone marrow fat content using FFMRI and hematological toxicity with patients undergoing concurrent chemoradiotherapy for cervical cancer. Carmona et al.^45^ similarly used FFMRI to quantify changes in bone marrow fat fraction as well as peripheral blood cell counts for patients undergoing pelvic chemoradiotherapy. Both studies found correlations between changes in bone marrow fat content and peripheral blood cells. In our study, not all patients received chemotherapy, and for those who did, complete blood data were unavailable. Blood count parameters, including white blood cell counts, hemoglobin levels, and platelet counts, have been shown to correlate with tumor response, inflammation, and overall treatment tolerance. Similar to Wang et al.^44^, %FF increased as the dose increased as shown in Figure 4. The mean delta for BM volumes was +16.9 %FF. Carmona et al.^45^ also observed an increase in %FF from baseline images and similarly concluded that dose leads to significant increases in %FF within the bone marrow. Conversely for MECT, we observed a decrease in mean CT number of −26.5 HU.

**FIGURE 5:**
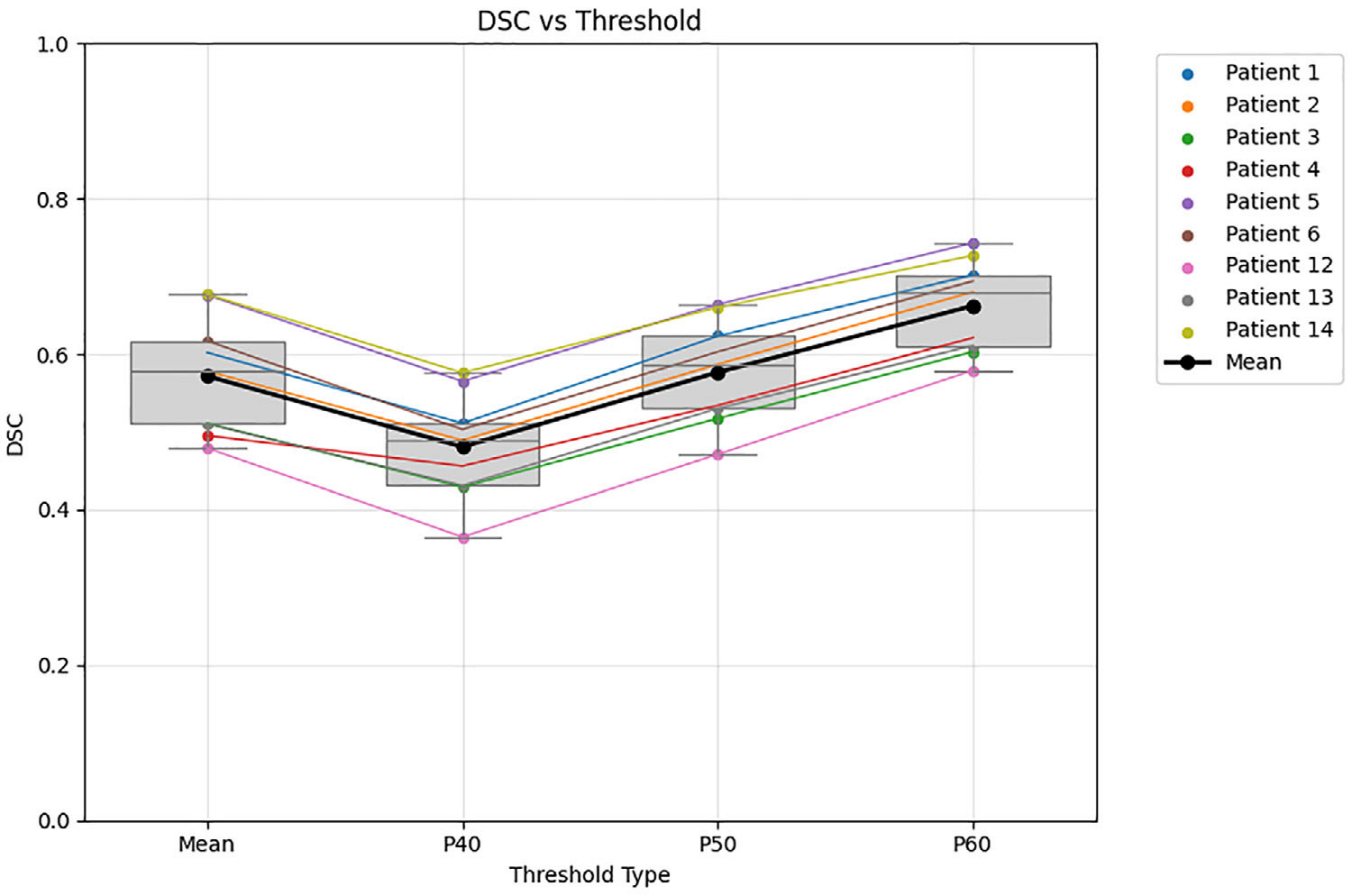
Dice similarity coefficient (DSC) as a function of threshold. A consistent increase in DSC with increasing threshold (P40 to P60) is observed across all patients, with the mean trend shown in black.

A threshold sensitivity analysis was performed to assess the dependence of the DSC on the threshold chosen for ABM definition, with full results provided in the Supplementary Material. A percentile‐based thresholding approach was derived from the 40^th^ (P40), 50^th^ (P50), and 60^th^ (P60) percentiles of the voxel intensity distribution within the total bone marrow contour. When compared between modalities, these volumes demonstrated a consistent monotonic increase in DSC across all patients as the volume increased, as shown in Figure 5. This behavior is expected as larger volumes increase the probability of spatial overlap between modalities. However, higher thresholds may also incorporate voxels that do not correspond to biologically active marrow, reducing specificity. The mean‐based threshold yielded intermediate DSC values within this range, supporting its use as a practical balance between overlap and biological relevance. Notably, mean‐based thresholding approaches have been used in prior functional marrow studies across CT, MRI, and PET modalities, including the work by Lawless et al.^20^ and others in bone marrow‐sparing radiotherapy workflows.^37^, ^38^, ^39^, ^40^, ^41^, ^42^, ^43^

**FIGURE 6:**
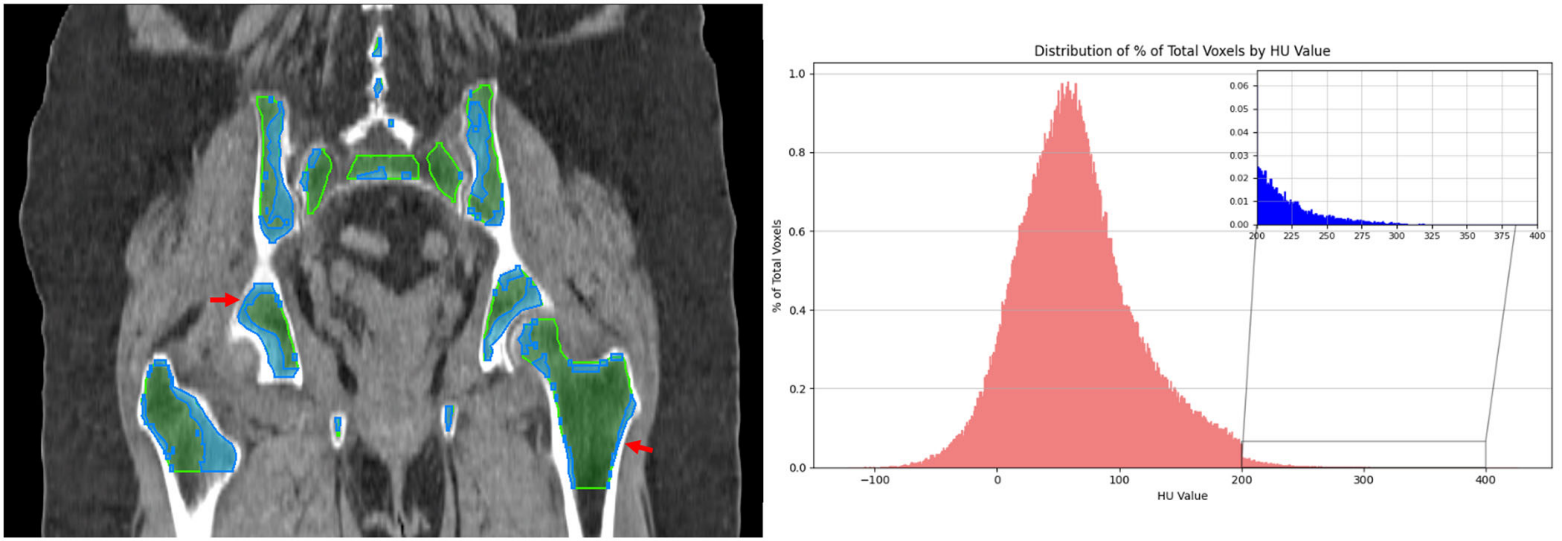
(Left) VNCa image showing the generated bone marrow contour (green) and ABM (blue). Arrows indicate regions where residual cortical bone signal is included in the bone marrow and ABM contour. (Right) histogram of the distribution of HU values within the bone marrow contour. The zoomed in plot shows a prominent high‐value tail indicating the presence of voxels with high HU values. These residual high HU values are attributed to incomplete calcium removal in the MECT image. Future work should identify if a larger contraction would be merited in these regions.

MECT produced larger ABM volumes than those derived from FFMRI on post‐treatment images as shown in Figure 3. We hypothesize that this difference is likely due to incomplete calcium removal in VNCa images, indicated by persistent high signal in cortical bone (Figure 1). When defining BM on VNCa images, these high‐density cortical regions remain even after marrow contraction. Consequently, attempts to contour ABM inadvertently include residual cortical bone along with the intended highly perfused, less fatty marrow regions. This inclusion leads to an overestimation of ABM volume by MECT methods as shown in Figure 6.

**FIGURE 7:**
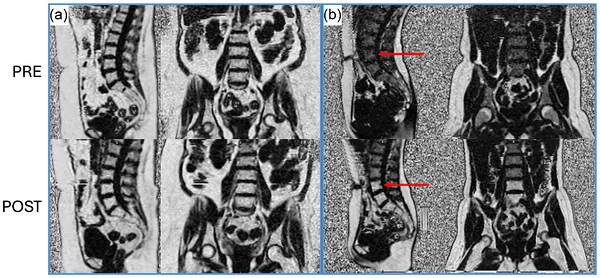
Pre‐ and post‐ treatment fat fraction MRI (FFMRI) images for (a) Patient 9 compared to (b) Patient 1. Patient 9 had a high initial %FF value for ABM compared to Patient 1. Patient 1 had a lower starting %FF but post‐radiation saw an increase in %FF as shown by the red arrows.

FFMRI does not rely on the removal of calcium to define the ABM, which in part leads to the smaller ABM volumes on FFMRI as compared to MECT. For all patients except Patient 9, a decrease in FFMRI ABM volume was seen between pre‐ and post‐radiation images. Patient 9 had the highest mean %FF value (75.78 %FF) for pre‐treatment ABM volume. We hypothesize that due to the high baseline fat fraction prior to treatment, the marrow was less susceptible to radiation induced changes compared to other patients. Figure 7 shows pre‐ and post‐treatment FFMRI scans for Patient 9 compared to those from Patient 1. The lumbo‐sacral spine in the pre‐ and post‐ FFMRI scans show a clear increase in %FF (brighter signal) for Patient 1. The mean ABM %FF value for FFMRI was 57.66 %FF, which is consistent with the findings from Lawless et al.^20^, who reported an average of 60 %FF across swine subjects.

**FIGURE 8:**
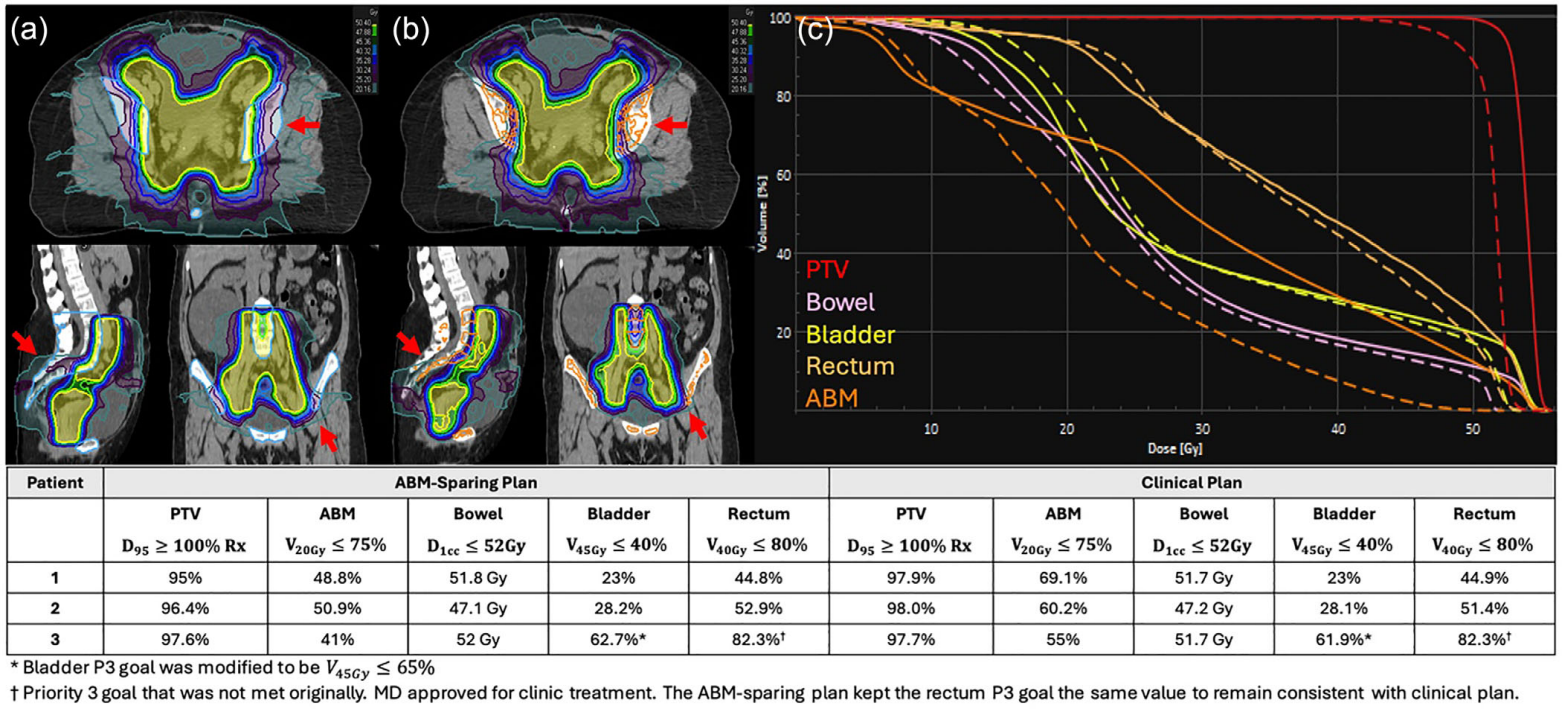
(a) An example of a clinical treatment plan without optimizing on ABM. The light blue contour includes all bony anatomy near the treatment site and is clinically defined as “bone marrow.” (b) The same patient but with an ABM‐sparing plan which was optimized on the ABM volume (orange contour) created from our study. (c) DVH comparing **clinical plan (—) and ABM‐sparing plan (‐—‐)**. All clinical goals were still met while optimizing based on ABM volumes as shown in the bottom table for three patients from the cohort.

McGuire et al.^9^ demonstrated a methodology to define regions of active bone marrow and create bone marrow sparing IMRT plans to reduce hematologic toxicities. Dinges et al.^46^ have expanded the use of pelvic bone marrow sparing to intensity modulated proton therapy (IMPT) as compared to IMRT showing promise that IMPT could further reduce hematologic toxicity when compared to IMRT. Both groups utilized [^18^F]FLT PET, which is a promising imaging technique for detecting cellular proliferation with demonstrated utility in bone marrow.^28^, ^29^, ^30^ However, FLT is not currently FDA‐approved as a radiopharmaceutical, restricting its use to clinical trials and preventing routine clinical implementation until regulatory approval is obtained. FFMRI and MECT offer a more accessible option for generation of ABM sparing treatment plans. This work supports the incorporation of these imaging modalities into the treatment planning process to enable ABM‐sparing radiation therapy, as illustrated in Figure 8. Example ABM‐sparing treatment plans were generated for three patients by incorporating ABM contours created from our technique into the optimization process and compared with the clinically delivered plan. Clinical objectives for target coverage ($D_{\mathrm{mean}}\geq$ D_50.4 Gy_, D_95%_ ≥ D_50.4 Gy_) and organs‐at‐risk (OAR) (bowel: D_1cc_ ≤ 52 Gy, V_40 Gy_ ≤ 20%; bladder: V_45 Gy_ ≤ 40%; rectum: V_40 Gy_ ≤ 80%) sparing were maintained while achieving improved ABM sparing relative to the clinically delivered plan. In our clinical workflow, all adjacent bony anatomy (from the lower lumbar spine through the femoral heads) is contoured and designated as “bone marrow.” The NCCN Guidelines Version 2.2026 for Cervical Cancer recommends a bone marrow dose objective of $V_{20Gy}\leq 75\%$.^47^ A future prospective study that implements ABM sparing using these methods while collecting hematologic data is needed to correlate HT with dose to the ABM.

Within the radiation therapy setting, MR scanners are increasingly available in RT clinics especially with MR‐only RT workflows remaining an active area of research and innovation.^48^ Integrating FFMRI into existing MRI protocols for cancer patients could allow for ABM data collection. Similarly, many modern CT simulation scanners now include MECT capabilities, enabling MECT acquisition during routine RT simulation. This work demonstrates that ABM delineation through more accessible MECT and FFMRI can be used not only to monitor changes in the marrow compartment, but also to generate ABM sparing treatment plans.

This feasibility study provides valuable insights into imaging and segmentation of ABM. However, several limitations should be acknowledged. First, the VNCa images demonstrated incomplete calcium removal, contributing to discrepancies in ABM volumes between MECT and FFMRI as shown in Figures 3 and 6. We believe that this incomplete calcium removal is a potential cause of discrepancies between the ABM volumes as determined by FFMRI versus MECT. While a uniform contraction was applied to the bony anatomy to reduce residual cortical bone signal, this approach may be insufficient in anatomically complex regions such as the pelvis. In these regions, thicker or irregular cortical bone can result in unwanted high‐density signal within the marrow compartment. Conversely, in regions with thinner cortical bone (e.g., vertebral bodies), the same margin may be too large and inadvertently exclude regions of potential ABM. This limitation was observed in the overestimation of MECT‐defined ABM volumes relative to FFMRI‐determined volumes. While qualitative examples (as seen in Figure 6) demonstrate residual cortical signal within ABM contours, the fraction of MECT‐defined ABM affected by incomplete calcium removal was not explicitly quantified in this study. Future work is merited to investigate the sensitivity of the ABM volume to this residual calcium signal. Improvements in MECT technology, such as photon‐counting detectors and added filtration, may help to mitigate this issue, but such technology was not available on the scanner used in this study. Second, registrations between datasets were performed using a per‐vertebral body rigid registration strategy. Each vertebral body was independently rigidly registered between MECT and FFMRI, and the resulting transformation was applied only to that specific vertebral body contour, rather than performing a single global registration over the entire pelvis. This approach was chosen to minimize the impact of inter‐scan differences in patient positioning and pelvic organ filling, which primarily affect soft tissues rather than rigid bony anatomy. While registration between MECT and FFMRI is intrinsically challenged by differences in image contrast, constraining the registration to individual vertebral bodies improves alignment robustness for bony structures. Registration uncertainty may also contribute to DSC findings. Because DSC reflects both biologic differences and registration uncertainty, the relative contribution of these factors to the observed overlap could not be determined. Residual registration uncertainty remains a limitation of this study and may contribute to measurement variability. Future work will focus on further refinement of multimodality image registration techniques, including evaluation of deformable image registration approaches for applications involving both bony and soft‐tissue anatomy.

Treatment‐related factors also introduced variability. Patients received different doses based on disease location and extent. For example, patients with disease in the para‐aortic lymph nodes had larger PTVs that extended superiorly along the lumbar spine. These variations in PTV size and delivered dose influenced the observed changes in ABM after treatment. As discussed, incorporating complete blood counts would have allowed us to explore potential associations between systemic changes and volumetric reductions observed on imaging, identify predictive biomarkers, and compare our findings to other studies. Future studies should integrate hematologic profiles to enable a more comprehensive assessment of treatment effects and patient outcomes. Finally, the relatively small sample size limits generalizability, and future studies with larger cohorts are needed to validate these findings. Future work should incorporate hematologic data to correlate ABM volume changes observed on MECT and FFMRI with systemic marrow suppression.

## 5 CONCLUSION

This study demonstrates the feasibility of identifying ABM using MECT and FFMRI imaging modalities and quantifying ABM volume changes after radiation therapy. By characterizing these changes, we provide evidence that ABM is substantially impacted by pelvic radiation, underscoring the importance of incorporating marrow‐sparing strategies into treatment planning. The ability to delineate ABM offers a pathway to integrate functional imaging into the radiotherapy workflow. This integration could support future work investigating the reduction of hematologic toxicity by sparing ABM while maintaining target coverage and organ‐at‐risk constraints. Future work should prospectively quantify dose‐response relationships between radiation exposure to ABM and hematologic toxicity. If a correlation is found, then this methodology could inform strategies to reduce hematologic toxicities, particularly for those receiving concurrent chemoradiation, where marrow preservation is critical. Future work should focus on validating these findings in larger cohorts, exploring automated segmentation methods for clinical implementation, and correlating imaging‐based ABM changes with hematologic biomarkers to strengthen the link between imaging and clinical toxicity. Ultimately, these advances could support the routine adoption of ABM‐sparing techniques in pelvic radiation therapy.

## AUTHOR CONTRIBUTIONS

**Carolyn Eckrich**: lead author, data acquisition and analysis, manuscript revision, and final review. **Kristin A Bradley**: patient identification, contouring, manuscript revision. **Bethany Anderson**: patient identification, contouring, manuscript revision. **Diego Hernando**: data acquisition, protocol refinement, manuscript revision. **Jessica R Miller**: conception of work, data analysis, manuscript revision. **Jainil Shah**: conception of work, CT protocol refinement, manuscript revision. **Patrick Wohlfahrt**: conception of work, CT protocol refinement, manuscript revision. **Michael Lawless**: conception of work, data acquisition and analysis, supervision, manuscript revision.

## CONFLICT OF INTEREST STATEMENT

This work was supported through funding provided by GE Healthcare and Siemens Healthineers. Jainil Shah and Patrick Wohlfahrt are employees of Siemens Healthineers. Unrelated to this work, Diego Hernando has ownership interests in Calimetrix, LLC.

## FUNDING INFORMATION

GE Healthcare research agreement involving Michael Lawless, Jessica Miller, and Kristin Bradley (not federal grant). Siemens Healthineers research agreement involving Michael Lawless and Jessica Miller (not federal grant).

## ETHICS STATEMENT

Ethical approval for this study was obtained from the IRB (approval number: UW22105).

## Supporting information



Supporting Information

## Data Availability

Authors will share data upon request to the corresponding author.
