# Supplementary material for: Feasibility of multi‐energy CT and fat fraction MRI for characterizing active bone marrow response to pelvic external beam radiation therapy
Source: J Appl Clin Med Phys. 2026 Sep 17;27(10):e70794. doi: 10.1002/acm2.70794 (PMC13585644; doi:10.1002/acm2.70794)
Supplement: Supplementary file 1 [file ACM2-27-e70794-s001.docx]

**Table 1.** Comparison of threshold values used to define active bone marrow (ABM) from MECT (HU) and FFMRI (%FF) imaging across patients. The Mean column represents the original thresholding method, in which the mean value of the total bone marrow contour was used to define ABM. The P40, P50, and P60 columns represent percentile-based thresholding approaches derived from the 40th, 50th, and 60th percentiles of the voxel intensity distribution within the total bone marrow contour.

|  | **MECT Threshold Value (HU)** | | | | **FFMRI Threshold Value (%FF)** | | | |
| --- | --- | --- | --- | --- | --- | --- | --- | --- |
| **Patient** | **Mean** | **P40** | **P50** | **P60** | **Mean** | **P40** | **P50** | **P60** |
| **1** | 65 | 72 | 61 | 51 | 36 | 32 | 36 | 39 |
| **2** | 40 | 46 | 33 | 19 | 59 | 53 | 56 | 60 |
| **3** | 15 | 24 | 8 | -6 | 61 | 55 | 57 | 62 |
| **4** | 6 | -17 | -2 | 13 | 71 | 68 | 72 | 76 |
| **5** | 3 | 17 | 2 | -12 | 66 | 58 | 64 | 71 |
| **6** | 75 | 83 | 72 | 60 | 42 | 33 | 37 | 42 |
| **12** | 32 | 40 | 23 | 8 | 56 | 48 | 52 | 55 |
| **13** | 33 | 43 | 27 | 12 | 62 | 55 | 60 | 65 |
| **14** | 5 | 19 | 5 | -9 | 64 | 56 | 61 | 68 |
| **Average** | 30 | 36 | 25 | 15 | 57 | 51 | 55 | 60 |

**Table 2.** Mean-based and percentile-based thresholding values used to create active bone marrow (ABM) volumes derived from MECT and FFMRI.

|  | **MECT ABM Volume (cc)** | | | | **FFMRI ABM Volume (cc)** | | | |
| --- | --- | --- | --- | --- | --- | --- | --- | --- |
| **Patient** | **Mean** | **P40** | **P50** | **P60** | **Mean** | **P40** | **P50** | **P60** |
| **1** | 323.8 | 280.2 | 350.5 | 417.5 | 358.6 | 272.5 | 355.5 | 424.2 |
| **2** | 239.8 | 217.3 | 267.4 | 324.7 | 294.1 | 206.0 | 260.3 | 318.1 |
| **3** | 309.8 | 274.0 | 341.4 | 405.7 | 353.8 | 255.0 | 308.6 | 374.3 |
| **4** | 279.6 | 252.0 | 311.9 | 375.9 | 293.6 | 248.8 | 306.7 | 368.4 |
| **5** | 387.7 | 314.9 | 393.7 | 472.1 | 388.6 | 298.7 | 366.4 | 441.3 |
| **6** | 280.1 | 240.2 | 297.3 | 360.8 | 352.2 | 236.0 | 297.1 | 363.98 |
| **12** | 312.7 | 281.3 | 351.3 | 422.5 | 410.7 | 277.6 | 336.4 | 392.9 |
| **13** | 286.5 | 234.8 | 293.1 | 350.5 | 300.1 | 224.8 | 289.0 | 340.2 |
| **14** | 325.8 | 257.9 | 322.9 | 386.1 | 342.2 | 251.4 | 313.7 | 377.7 |
| **Average** | 305.1 | 261.4 | 320.4 | 390.6 | 343.8 | 252.3 | 314.2 | 377.9 |

**Table 3.** Dice similarity coefficient (DSC) comparing spatial overlap of ABM volumes generated using mean-based and percentile-based thresholding methods.

|  | **DSC** | | | |
| --- | --- | --- | --- | --- |
| **Patient** | **Mean** | **P40** | **P50** | **P60** |
| **1** | 0.60 | 0.51 | 0.62 | 0.70 |
| **2** | 0.58 | 0.49 | 0.59 | 0.68 |
| **3** | 0.51 | 0.43 | 0.52 | 0.60 |
| **4** | 0.50 | 0.46 | 0.53 | 0.62 |
| **5** | 0.68 | 0.57 | 0.66 | 0.74 |
| **6** | 0.62 | 0.50 | 0.60 | 0.69 |
| **12** | 0.48 | 0.37 | 0.47 | 0.58 |
| **13** | 0.51 | 0.43 | 0.53 | 0.61 |
| **14** | 0.68 | 0.58 | 0.66 | 0.73 |
| **Average** | 0.58 | 0.48 | 0.58 | 0.66 |
